# Male Antarctic fur seals: neglected food competitors of bioindicator species in the context of an increasing Antarctic krill fishery

**DOI:** 10.1038/s41598-020-75148-9

**Published:** 2020-10-28

**Authors:** A. D. Lowther, I. Staniland, C. Lydersen, K. M. Kovacs

**Affiliations:** 1 Norwegian Polar Institute, Tromsö, Norway; 2 British Antarctic Survey, Cambridge, UK

**Keywords:** Animal migration, Behavioural ecology, Conservation biology, Ecosystem services

## Abstract

The fishery for Antarctic krill is currently managed using a precautionary, ecosystem-based approach to limiting catch, with performance indices from a long-term monitoring program focused on several krill-dependent predators that are used to track ecosystem health. Concerns over increased fishing in concentrated areas and ongoing efforts to establish a Marine Protected Area along the Peninsula, a key fishing region, is driving the development of an adaptive management system for the fishery. The cumulative effects of fishing effort and interactions among krill-dependent predators and their performance is at present neglected in the CCAMLR Ecosystem Monitoring Program. However, we show considerable overlap between male Antarctic fur seals and the krill fishery in a complex mosaic, suggesting potential for cumulative impacts on other krill dependent predators. A holistic view is required as part of future efforts to manage the krill fishery that incorporates various sources of potential impacts on the performance of bioindicator species, including the fishery and its interactions with various krill dependent predators.

## Introduction

Climate modifications to marine ecosystems continue unabated, accompanied by pressures to maintain ecosystem services such as fishing. This challenging combination has led to considerable concern as to how to balance resource exploitation and ecosystem resilience and health. Marine ecosystems must ideally support the needs of the guild of marine predators that rely on a resource and predation by commercial fisheries which target the same resource. Knowledge regarding the spatial and temporal overlap between natural predators and fisheries is a precursor to determining the degree and significance of any functional overlap, which can then be prioritised for management and mitigation measures, forming the basis of Ecosystem Based Fisheries Management (EBFM)^1,2^.

In the western Antarctic Peninsula (WAP) and Scotia Sea the diet of upper trophic marine predators is heavily dominated by Antarctic krill *Euphausia superba*, a keystone species in a short-chain food web typical to polar regions^3^. Historically, this region has undergone substantial modification to the food web, with uncontrolled harvesting of krill-eating whales, seals and mesopelagic fishes throughout the Southern Ocean during the nineteenth and early twentieth century. While upper trophic harvesting ended by the latter half of the twentieth century, the impacts on the WAP and Scotia Sea ecosystem from the extreme and rapid depletion and subsequent variable levels of recovery are unknown. The WAP is also undergoing rapid and nonlinear restructuring due to the impacts of a warming environment, with areas responding differently contingent on the interaction between large scale and fine scale processes interlinking meteorological, hydrographic, sea ice and primary productivity variability (e.g. Schofield et al.^4^). Amidst this incredibly complex and altered marine ecosystem operates the largest volume fishery in the Southern Ocean. The fishery for Antarctic krill, henceforth krill, is a mid-water trawl fishery with a catch that has increased recently to approximately 300,000 tonnes per annum. The fleet used traditional (TR) trawl gear exclusively until 2006, whereupon a continuous pumping (CP) system initially tested by Vanuatu in 2004 was introduced into the fishery by Norway^5^. The more modern CP trawlers are thought to be more efficient and some believe that they have a lower ecological impact than TR trawling due to their smaller ecological footprint, though this has not been empirically demonstrated^5^. The Commission for the Conservation of Antarctic Marine Living Resources (CCAMLR) manages the fishery in a precautionary fashion, by setting a Total Allowable Catch Limit of 5.6 million tonnes. However, a trigger level of 620,000 t has been set because of a lack of scientific data to ensure the fishery is precautionary. In 2009, CCAMLR introduced measures designed to reduce the spatial concentration of the catch by allocating a percentage of the trigger level to different Subareas. Furthermore, CCAMLR has mandated that the trigger level will remain in place until such time that ecosystem-based fisheries management (EBFM) strategies can be employed that will avoid harvest levels that might impact the predator–prey balance and cause irreversible damage to the marine ecosystem. Efforts to characterise the spatial footprint of the fishery have been limited to kernel density summaries of summed fishing effort over decadal time periods (e.g.^6,7^) with the exception of one study that identified temporally persistent fishing hotspots over six consecutive seasons^8^. Interestingly, this study proposed that the fishery should be considered another member of the krill predator guild.

Juxtaposed with efforts to achieve EBFM is the recent development of a proposal for a Marine Protected Area^9^ in the WAP region, comprising a suite of statically-bounded zones with a variety of different management measures. Integrating the two strategies presents a complex managerial challenge for CCAMLR; efforts to develop EBFM for the krill fishery have been underway for over 2 decades, with little progress in achieving a viable, practical solution. Several approaches have been proposed previously, all of which rely to some extent on performance signals derived from a 30+ year time series of krill predator monitoring that was initiated by CCAMLR in 1987^10^.

The CCAMLR Environmental Monitoring Program (CEMP) currently monitors breeding krill-dependent predators, with efforts in the western Antarctic Peninsula being dominated by penguin studies. CEMP monitoring studies on Antarctic fur seals (AFS: *Arctocephalus gazella*) focus exclusively on adult females and their pups, on populations in the South Shetlands and South Georgia Islands. However, male AFS are a numerically abundant krill predator that is likely capable of significant competitive pressure with respect to CEMP monitored species such as chinstrap penguins. Male AFS provide no parental care to their offspring and move southwards to the South Orkney Islands, presumably to access higher quality foraging grounds once mating is complete at South Georgia^11^. Indeed, census data from the South Orkney Islands as far back as the late 1970’s describe a rapid increase in numbers of juvenile, subadult and nonbreeding adult male AFS^12^ during late January. The seasonal influx of potentially vast numbers of spatially unconstrained krill predators that are unaccounted for in current assessments could have implications for disentangling the key drivers of ecosystem processes. In the context of the WAP, these processes are often characterised by models linking predator performance indices, large-scale climate processes and fishing effort^13–16^ with many being underpinned by model parameter estimates that neglect male AFS below reproductive age^17^.

On the other hand, breeding pygoscelid penguins, such as chinstrap penguins *Pygoscelis antarcticus* are at their most spatially and temporally constrained in terms of at-sea foraging behavior between mid-January and mid-March. Adults must return to land frequently in order to feed growing chicks. For penguins, that must feed their young with prey items directly, this means foraging trips that last only a few hours, with adults often traveling less than 50 km to find food^18^.

Our study aims to characterise the four-dimensional movement characteristics of a three-predator guild of unconstrained male AFS, CP and TR trawlers. Specifically, we quantify the overlap between this predator guild in relation to potential cumulative impacts on a representative CEMP monitored predator, chinstrap penguins. We hypothesise that male AFS overlap in both time and space with the fishery and that the two fishing gear types have different spatiotemporal patterns, resulting in a mosaic of potential competitive pressures. Finally, we discuss these competitive pressures in the context of interpreting performance indices of CEMP monitored predators as bioindicators of fishing pressure.

## Materials and methods

Field work for our study was conducted at Powell Island in the South Orkney Islands during the austral summer of 2015–2016 (henceforth 2016, Fig. 1). Twenty juvenile, subadult and adult male AFS were instrumented with either Sea Mammal Research Unit (SMRU) low-profile satellite relayed data loggers (SRDL, N = 18) that transmitted 6 hourly summary dive data in addition to locations, or SMRU Conductivity, Temperature and Depth loggers (CTD-SRDL, N = 2), which in addition to dive summary data also sent randomly selected CTD profiles daily^19^. Individual male AFS were remotely injected with an intramuscular anaesthetic (Zoletil^©^) at a dosage of 1.48 mg/kg estimated body mass, using a Paxarms modified 0.22 LR dart gun. Individuals who were not sufficiently drugged for safe handling were treated with a gaseous anaesthetic (Isofluorane^©^) at a 5% induction/0.5–1.5% maintenance level via a portable closed-circuit anaesthetics’ machine (Stinger, Advanced Anaesthesia Specialists, Sydney Australia). Electronic tags were attached on the dorsal pelage at a point midway between the foreflippers, using a two-part epoxy resin (Araldite 2012^©^).

**Figure 1:**
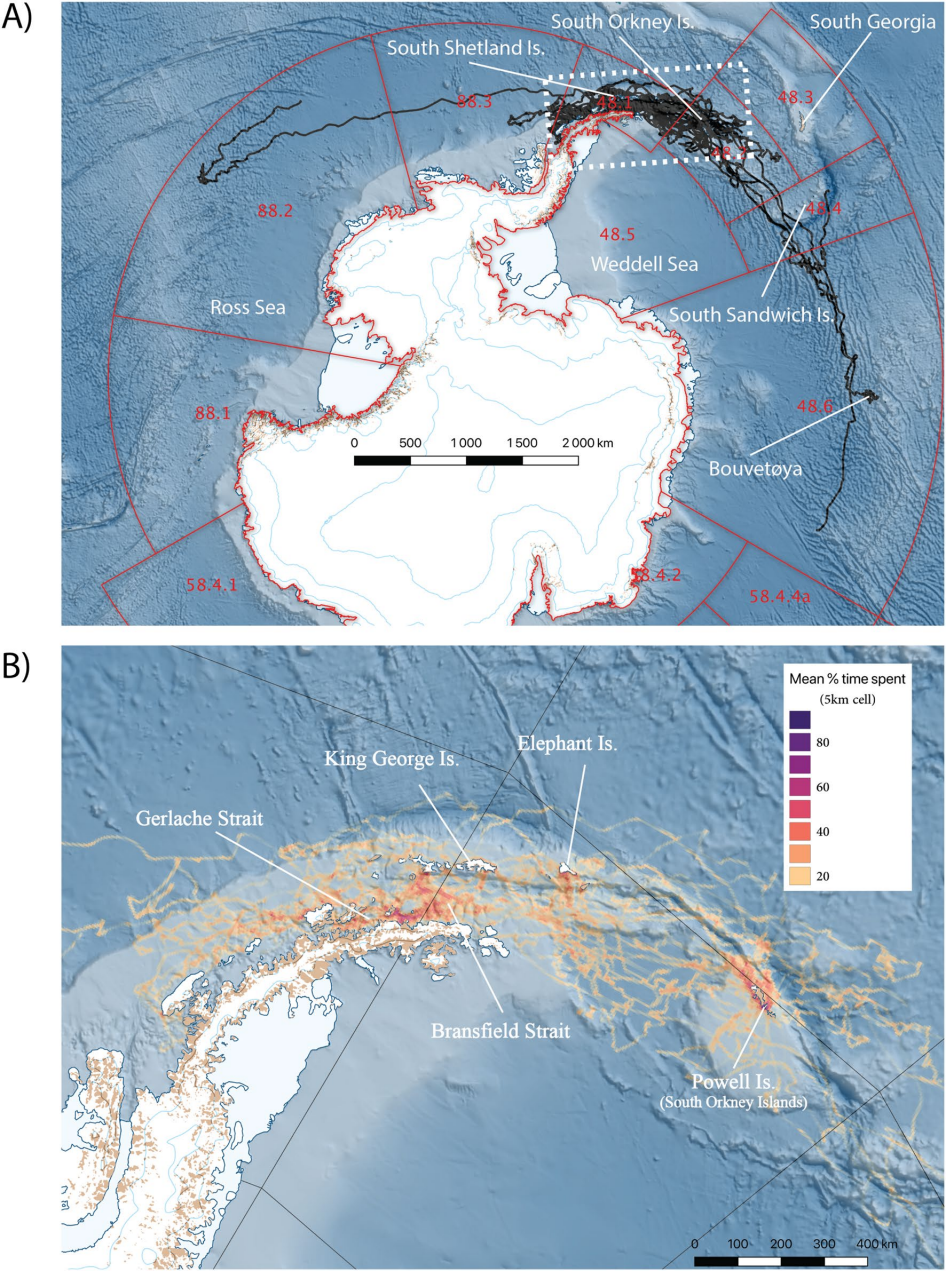
(**A**) Argos satellite telemetry data from 20 adult male Antarctic fur seals between 1st January and 8th December 2016 tagged at Powell Island, South Orkney Islands. The seals were fitted with either low profile Satellite Relay Data Loggers (SRDL) with incorporated dive loggers (N = 18) or Conductivity-Temperature-Depth SRDL (N = 2).Two animals ranged as far as the eastern Ross Sea and east of Bouvetøya in the Atlantic, though the remaining 18 individuals remained within the Scotia Sea and western Antarctic Peninsula. Our study focussed on the 18 animals that remained in the key krill fishing grounds (white dashed box) within CCAMLR Statistical Subareas 48.1 and 48.2 (shown in red). (**B**) The most probable at-sea locations from each animal were interpolated every 2 h within a continuous state space movement model framework for each animal. Filtered location data were then subsequently fitted with a Biased Random Bridge to estimate the 95% Utilisation Distribution (UD) across the entire dataset. Data are presented as a heatmap of intensity distribution reflecting the average time spent by animals in 5 km resolution grid cells. The figure was prepared using Quantarctica 3.12 (www.npolar.no/quantarctica)^59^.

### Male AFS instrumentation data

Both electronic tag variants were set to transmit a signal to the ARGOS CLS satellite constellation every 40 s while at sea, providing a doppler-based estimate of location post-processed by a Kalman filter by the data provider to accuracies ranging from 250 m—10’s of km^20^. When tags were dry (for a predetermined period of greater than 4 h), transmission rates shifted to every 80 s for two of every 5 h, until it was again immersed in seawater, thus providing the means to partition the location data temporally for each individual between at-sea and hauled out on land. Each tag variant also recorded data on each dive performed, then processes these data onboard and transmitted 6 h summaries. Summary data included the number of dives conducted (a dive being defined as deeper than 2 m and longer than 8 s), mean and maximum dive depths and dive time (the time taken from departing the surface until returning to it). Only days with complete summary data available (i.e. four consecutive six hourly summaries) were used for further analysis.

SRDLs also recorded the water temperature throughout a subset of dives (as a minimum). If a dive is one of the two deepest in a given two-hour period, the temperature was recorded at 1 s intervals on the ascent phase. The temperature and depth data were then processed onboard and summarized using the broken-stick method used in XBT casts, resulting in 12 temperature-depth points along the ascent of each dive profile. The two CTD-SRDLs utilize the same broken-stick method of onboard data compression, however these tags select the deepest CTD profile in the same period and provide the salinity, temperature and depth data at intervals along the ascent.

### Antarctic krill fishery data

The Antarctic krill fishing season starts and ends in November and thus spans parts of two calendar years—we henceforth refer to the fishing season by the year in which it closed. We used the CCAMLR C1 Antarctic krill Catch and Effort dataset to summarize catch distribution for continuous pumping (CP) and traditional (TR) trawlers for the 2016 fishing season and over the combined dataset spanning 2009–2018.

### Statistical analyses

#### Male AFS instrumentation data processing

Raw telemetry data were handled in two stages. Firstly, locations recorded by the instruments during haulout (i.e. on land) were removed so that the remaining telemetry data represented only at-sea bouts for each individual. These locations were subsequently processed using a continuous-time correlated random walk model^21^, using empirically derived error estimates for each Argos CLS location class to create a continuous movement path model, from which location predictions were made at 2 h intervals. Given that telemetry processing methods still have a level of uncertainty regarding the true location of an individual, we choose to characterise marine habitat usage by constructing Utilisation Distributions (UD) of home range (95%) and core areas (25%). We applied Biased Random Bridges to the modelled, predicted locations to account for serial temporal autocorrelation inherent in movement paths^22^ at a resolution of 5 km to account for the remaining, post-processed uncertainty in animal locations^20^. We then visualized habitat UD as a function of Intensity Distribution (the residency time in hours within 5 km grid cells) by calculating the mean cell values across all 20 individuals.

Dive data summarized at six-hourly intervals were used as dependent variables in a series of Generalised Additive Mixed Models (GAMM), with time period nested within Julian Day (to account for multiple time periods in a 24 h period) as a fixed effect and each individual animal as a random effect. To place dive data into context, environmental data in the form of daylight length and water temperature were used. Daylight length at Deception Island (60.57° W, 62.95° S) was calculated for each day of tracking using the R package “solarPos”. Spatio-temporal water temperature profiles derived from the temperature sensors on each instrument were visualized with Ocean Data View 4.7.8^23^ using the Data Interpolating Variational Analysis (DIVA) gridded interpolation software over a 500 km section stretching from the eastern Bransfield Strait to the bottom of the Gerlache Strait. Water column temperature profiles were then characterized between January and March (summer) and April to June (autumn/winter).

#### Antarctic krill fishery data

To spatially represent the fishery, we used the reported location at which each tow was set and assumed that this represented the true location (that is, without spatial error). We first applied a 95% Gaussian kernel weighted by catch (in tonnes) at a 5 km resolution with a 10 km search range, representing the approximate distance of a TR trawler haul and matching the resolution of the male AFS treated telemetry data^24^. As the fishery changed its location throughout the season and given that the fishery in Subareas 48.1 and 48.2 is orientated broadly along an east–west axis, we characterized its temporal evolution by the longitudinal movement of the two fishing gear types over time. Finally, to facilitate comparison with the vertical exploitation of habitat by male AFS, we describe the temporal change in reported trawl depths for each gear type over the season.

#### Spatio-temporal overlap between male AFS, fishing gear types and chinstrap penguins

Using the generated 5 km resolution rasters for the 2016 season fishing catch and male AFS distribution, we calculated the area (km^2^) and proportional overlap of UD between male AFS at 25% (core areas) and 95% (home range) with the 95% kernel of gear types. Information on the modeled distribution of chinstrap penguin foraging habitat throughout the study area is available in^6,7^ which, when placed into the context of our study, suggests there is considerable spatial overlap across the male AFS—gear type—chinstrap penguin predator guild. To extend this understanding, we incorporated the level of temporal overlap among the various krill fishers by comparing the longitudinal displacement of individual male AFS and each fishing gear type over time to the median fledging date of chinstrap penguins^25,26^. All analyses were conducted in R and are presented as mean (± Standard Error) unless otherwise stated.

### Ethical statement

All experimental research was carried out in accordance with the guidelines and regulations and permitted by the British Antarctic Survey Animal Welfare and Ethical Review Body (2015).

## Results

### Male AFS instrumentation data

**Figure 2:**
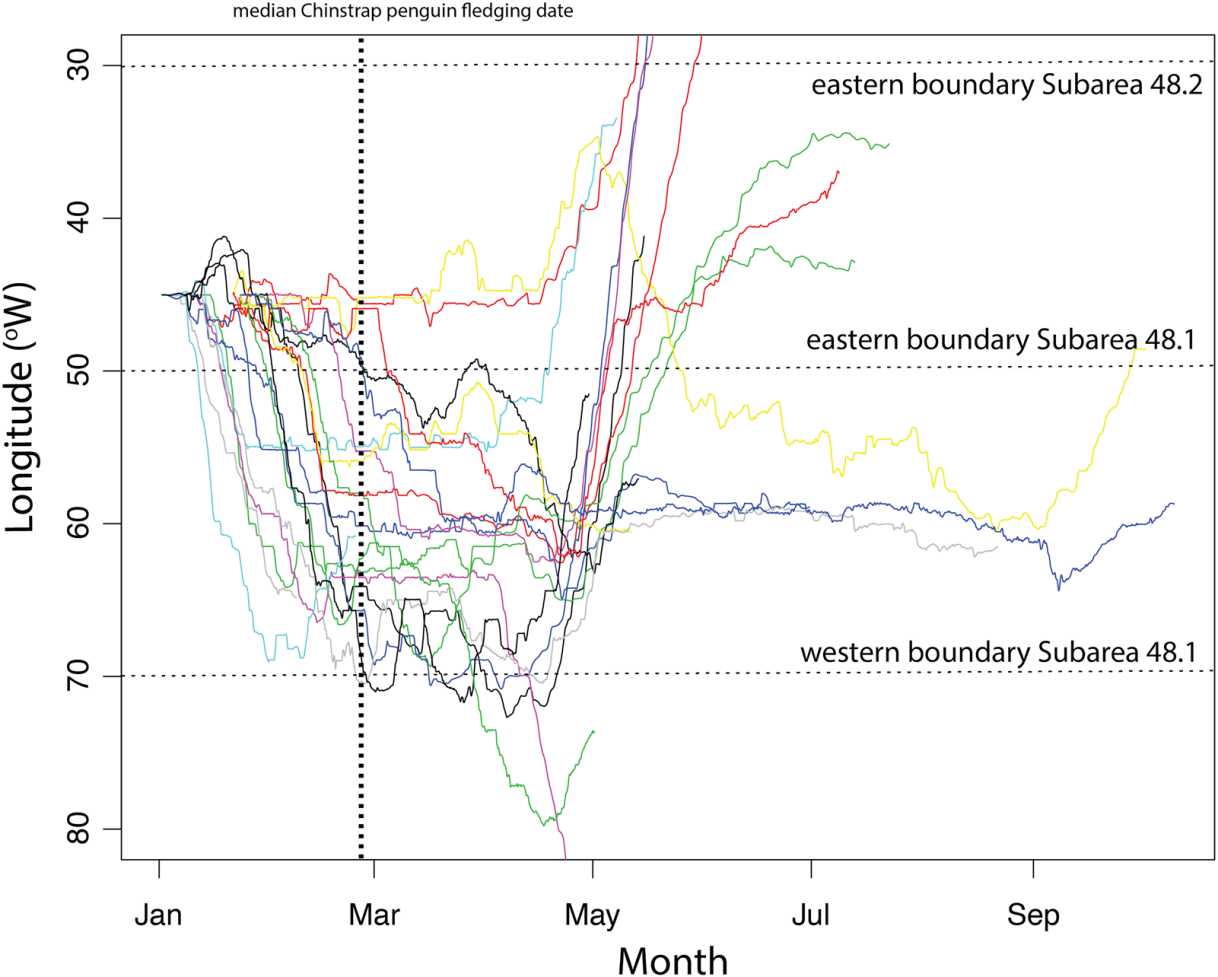
Longitudinal position of individual male Antarctic fur seals throughout the tracking period. The vertical dashed line represents the median date of fledging of chinstrap penguins in the Bransfield Strait (26th Feb^38^) and the Scotia Sea (2nd Mar^39^). Horizontal dashed lines bracket the key region in which the krill fishery operates, between the eastern boundary of Subarea 48.2 (30° W) and the western boundary of Subarea 48.1 (70° W). At the median chinstrap penguin fledging date, the fishery had taken ~ 18% of the Subarea 48.1 trigger level of 155,000 t (CCAMLR^34^). The majority of male Antarctic fur seals had moved into Subarea 48.1 by late January and remained in the Bransfield Strait throughout the key fishing period (April–May).

Induction times for immobilisation of male AFS ranged between 6 and 29 min; once inducted all procedures were completed within 23 min (18.8 ± 0.78 min) (Supplementary Table 1). The seals ranged in body length from 127 to 184 cm (mean 160.9 ± 3.71 cm; Supplementary Table 1), reflecting an age range from 3- > 8 year of age^27^. Instrumented individuals provided telemetry data for between 37.5 and 260 days (mean 166 ± 16.54 days), with a mean 1119 ± 172 raw location estimates over 2–23 at-sea bouts of 4–60 days duration, interspersed with haulouts that were less than 3 days long (mean 0.6 ± 1 day; Table 1. Adult male AFS ranged over large distances, reaching as far west as the eastern Ross Sea and east to Bouvetøya, though most individuals stayed between the South Orkney Islands and the Gerlache Strait (Fig. 1). While some males stayed resident around the South Orkneys throughout the entire study, many moved into the Bransfield Strait in late January/early February, with some remaining in the strait until just before the subsequent breeding season on South Georgia (Fig. 2).

**Table 1:** Summarised deployment, movement and dive data for 20 adult male Antarctic fur seals instrumented with either low profile Satellite Relay Data Loggers (SRDL) with integrated dive loggers (N = 18) or Conductivity-Temperature-Depth SRDL (N = 2) between 5th and 26th January 2016 at Powell Island, South Orkney Islands. Dashed lines represent instruments that failed to collect dive data.

ID	Date	Deployment (d)	Trips (N)	Duration (days)				Dives (N)	Dive depth (m)			Duration (min)		
				Foraging trip	± SD	Haulout	± SD		Max	Mean	± SD	Max	Mean	± SD
ag01-01-15	05/01/2016	127.2	9	12.76	22.24	0.7	0.55	-	-	-	-	-	-	-
ag01-02-15	06/01/2016	163.7	8	18.68	24.11	0.56	0.54	10,282	155	16.45	7.93	4.8	1.55	1.45
ag01-03-15	06/01/2016	191.7	23	4.06	6.21	2.6	2.75	-	-	-	-	-	-	-
ag01-04-15	07/01/2016	171.8	9	25.1	20.14	0.49	0.49	18,393	215	21.09	11.28	6.13	1.79	1.54
ag01-06-15	08/01/2016	126.9	13	6.98	9.97	0.83	0.91	16,243	187.5	17.77	8.35	5.87	1.54	1.39
ag01-07-15	08/01/2016	225.3	12	21.34	44.83	0.56	0.63	17,448	90	12.19	5.85	5.07	1.35	1.27
ag01-08-15	09/01/2016	254.8	15	20.69	33.24	0.55	0.54	22,572	180	16.04	9.25	6.4	1.67	1.47
ag01-09-15	09/01/2016	37.5	2	15.64	0.19	0.45	0.52	3817	157.5	15.02	6.84	4.27	1.6	1.34
ag01-10-15	10/01/2016	127.7	2	59.81	59.81	0.41	0.36	13,677	270	17.23	8.37	24.13	1.83	2.25
ag01-11-15	10/01/2016	172.1	7	46.66	55.27	0.6	0.66	9945	142.5	14.95	7.45	5.33	1.1	1.06
ag01-12-15	11/01/2016	171.5	7	36.96	48.16	0.45	0.43	16,624	187.5	15.94	9.98	5.33	1.58	1.28
ag01-13-15	11/01/2016	200.6	11	21.63	31.49	0.53	0.55	24,939	160	18.84	8.86	5.6	1.79	1.37
ag01-14-15	21/01/2016	54	2	15.65	3.03	0.96	3.37	1155	90	3.98	4.17	3.47	0.44	0.84
ag01-16-15	21/01/2016	213.5	7	36.9	68.35	0.45	0.67	14,572	147.5	13.37	8.69	4.53	1.34	1.07
ag01-17-15	21/01/2016	109.4	7	12.08	10.89	0.47	0.38	11,994	105	9.5	5.46	3.2	0.93	0.9
ag01-18-15	21/01/2016	223.1	12	25	39.94	0.6	0.53	23,015	185	21.48	9.56	6.4	2.28	1.71
ag01-19-15	23/01/2016	119.2	11	9.54	6.5	0.79	0.66	-	-	-	-	-	-	-
ag01-20-15	23/01/2016	169.2	10	14.81	24.26	0.41	0.58	31,819	150	14.86	7.03	11.33	1.51	1.29
ag01-244g-14	26/01/2016	260.6	10	13.13	15	0.86	1.14	6376	120	14.52	5.5	4.53	1.39	1.28
ag01-273-14	26/01/2016	98.9	13	18.57	21.25	0.67	0.81	23,318	280	43.83	13.72	7.2	2.81	2.14

**Figure 3:**
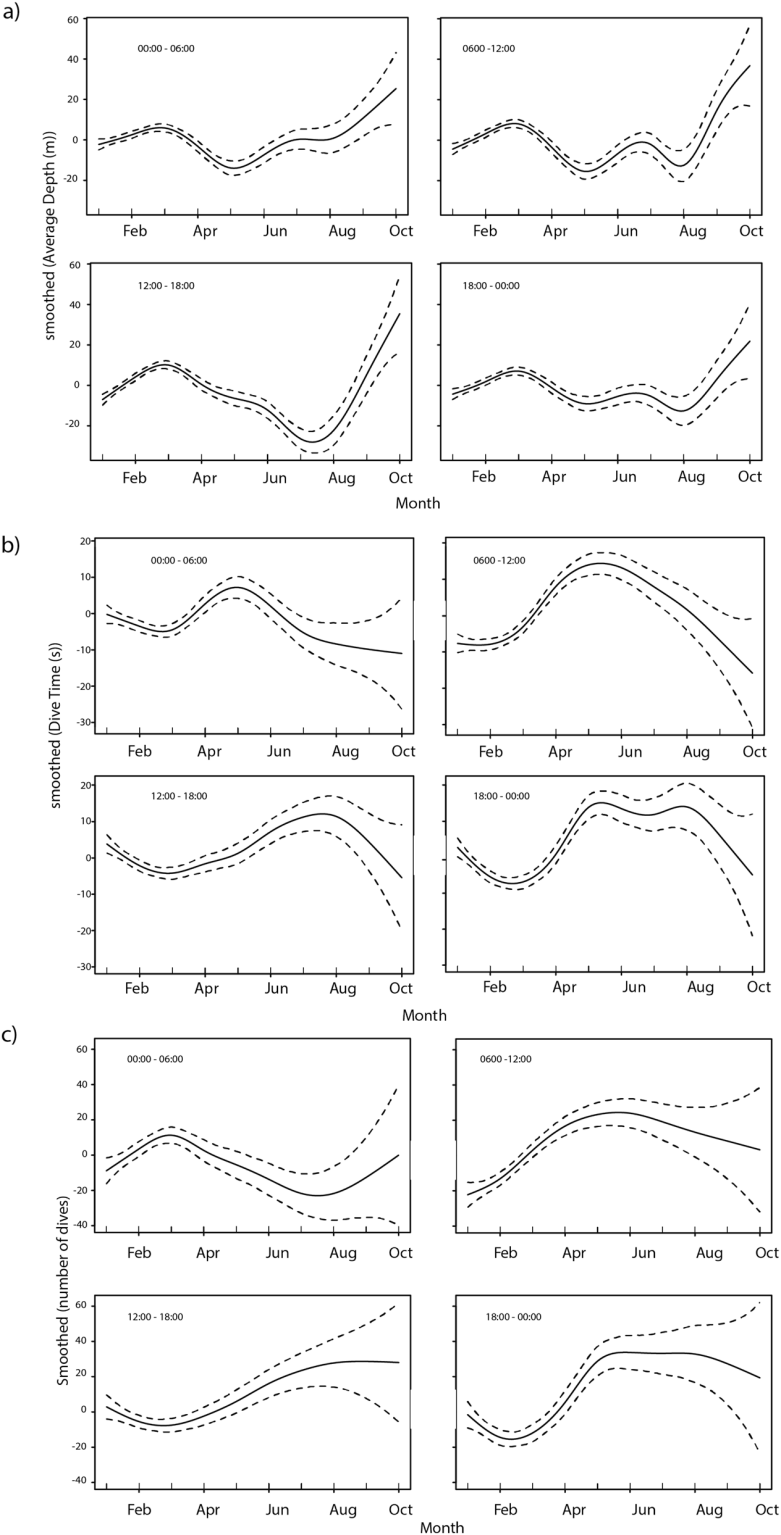
Smoothed responses from Generalised Additive Mixed Models of the (**A**) average dive depths, (**B**) dive time and (**C**) number of dives within 6 h time periods (00:00–06:00, 06:00–12:00, 12:00–18:00, 18:00–00:00) each day across all individuals. There was a consistent trend of increased average dive depth and dive time between April and August and a concomitant decrease in the number of dives between 0000 and 06:00.

Instruments from three of the seals failed to provide meaningful dive data, but from the other 17 individuals a total of 266,189 dives were collected (range 3817–31,819 dives per individual, mean 13,365 ± 2068) (Table 1). Mean maximum and average dive depths were 166 m (± 53.9 range 90–280 m) and 16.6 m (± 8.1, range 3.9–43.8 m), respectively. Average dive duration was 1.6 min (± 0.52, range 0.44–2.81 min) (Table 1). At the start of the instrumentation period, average and maximum dive depths were shallow (100 m and 110 m, respectively), despite near-constant daylight, with dives to only 50 m or less being common in February and March (Supplementary Fig. 2). As autumn progressed into winter and day length decreased, dives became deeper with animals diving to maximum depths of 200 m regularly (Supplementary Fig. 2). These patterns are supported by the smoothed response of dive parameters as the study period progressed, characterizing a trend of increased number of dives as well as increased average depth and duration, particularly from dawn to late evening though between midnight and early morning these patterns were reversed (Fig. 3). This progression to deeper diving at the onset of winter was matched by the behaviour of both fishing gear types, which also shifted to deeper fishing within the same vertical range as the male AFS (Supplementary Fig. 3). The change in exploitation depth matched changes in the physical structure of the water column, with temperature profiles from male AFS SRDL showing the upper 100 m of the water column to be between 0° and + 1 °C during the summer, cooling down to below zero during the winter (Supplementary Fig. 4).

### Spatio-temporal overlap between male AFS, fishing gear types and chinstrap penguins

Some male AFS dispersed over vast oceanic areas, though most of the animals remained within the WAP and Scotia Sea (Fig. 1). Typically, male AFS remained at Powell Island for up to two weeks after instrumentation, though some stayed within the South Orkneys until the end of June. They were likely forced away from the islands by thickening sea ice^28^. However, most of the instrumented animals moved into the Bransfield Strait by late January and remained there until the transmitters stopped functioning in late winter/early spring.

The 95% UD of all males (derived from Biased Random Bridges) covered an area an order of magnitude greater than the fishery in 2016 and more than twice the size of the area exploited by trawlers between 2009 and 2018 (Table 2). The areas exploited by both gear types occupied less than 6% of male AFS home range during the study, though up to 18% of the recent historical area of the fishery (Table 2). Conversely, the majority of the area exploited by both fishing gear types was within the range of the male AFS. The core 25% UD of male AFS was focused in the southern central Bransfield Strait, the southern aspect of Elephant Island, and around the South Orkney Islands coastal shelf (Figs. 1b, 3a; Table 2), overlapping between 25 and 28% of fished areas depending on fishing gear type (Table 2).

**Table 2:** Area and the proportional overlap between the 95% Utilisation Distributions (UD) of male Antarctic fur seals (male AFS), continuous pumping (CP) and traditional trawling (TR) in 2016 and across the entire 10 year dataset (2009–2019) and the combined efforts of the fishing fleet for 95% UD. Numbers in parentheses represent similar comparisons of areal size and propotional overlap at the 25% UD core range of mAFS. Data above the diagonal are overlaps of column-within-row and vica versa for below the diagonal; for example 74% of the continuous pumping trawl home range were contained within that of male AFS home range, yet only 5% of male AFS home range fell within areas exploited by the pump trawlers. Note the disproportionate levels of overlap with male AFS core range, suggesting that while they utilised a much broader home range than the fishery, the same areas were preferred by both.

	95% (25%) area (km^2^)	Proportional overlap 95% (25%) UD					
		Male AFS	CP (2016)	TR (2016)	CP (all years)	TR (all years)	Fishery (all years)
Male AFS	194,225 (22,250)	–	0.74 (0.26)	0.69 (0.28)	0.7 (0.24)	0.51 (0.14)	0.53 (0.15)
CP (2016)	12,375	0.05 (0.14)	–	0.27	0.34	0.14	0.16
TR (2016)	18,250	0.06 (0.23)	0.4	–	0.31	0.25	0.22
CP (all years)	32,938	0.11 (0.36)	0.91	0.56	–	0.38	0.42
TR (all years)	69,554	0.18 (0.43)	0.79	0.95	0.8	–	0.77
Fishery (all years)	72,704	0.19 (0.51)	0.92	0.89	0.92	0.8	–

**Figure 4:**
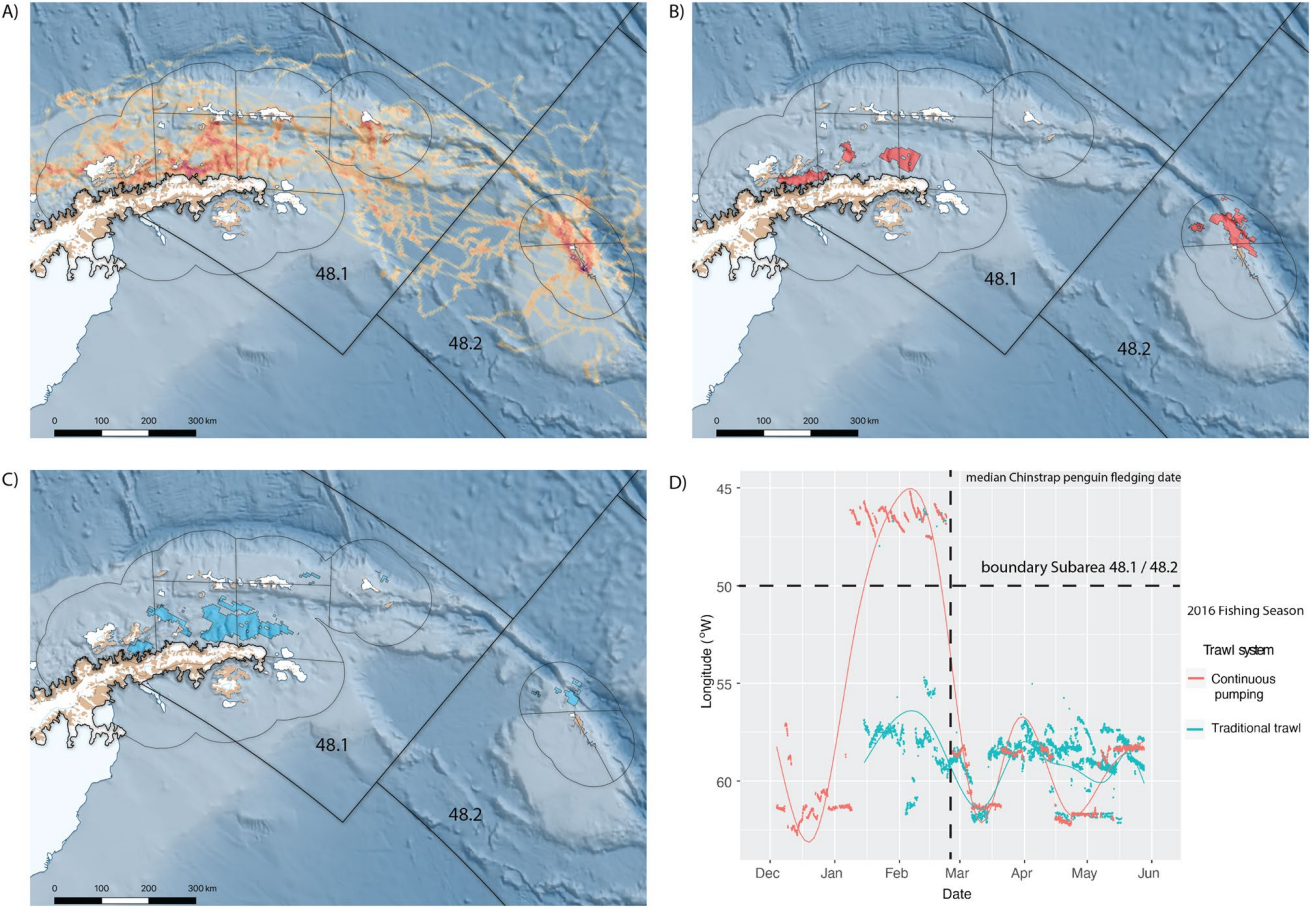
(**A**) At-sea distribution of mean time spent within 5 km grid cells of male Antarctic fur seals (AFS) instrumented at the South Orkney Islands in 2016 that remained within the broad western Antarctic Peninsula and Scotia Sea region (N = 18). The overlap between the 95% Utilisation Distribution home range of male AFS and those of the commercial krill fishery operating, (**B**) continuous pumping and, (**C**) traditional trawling fishing gear are shown. Thick black lines indicate the CCAMLR Statistical Subareas 48.1 and 48.2 (labelled) while thinner lines show the Small Scale Management Units (SSMU) originally considered for managing the fishery at smaller than Subarea scales. (**D**) Temporally there was a pronounced difference in how the fishery was distributed contingent on what gear was used, with continuous pumping trawlers (red dots and curvilinear line) beginning the season in the Bransfield and Gerlache Straits before fishing off the western end of the South Orkney Islands between January–March and subsequently moving back into the Bransfield Strait until late May/early June. In contrast, vessels using traditional trawls (blue dots and curvilinear line) fished within the Bransfield and Gerlache Straits from mid January until their departure in late May. Thus, overlap between fishing vessels and male AFS foraging varied both spatially and temporally depending on the fishing gear being used. The median fledging date of Chinstrap penguins is included here to facilitate comparisons of spatiotemporal overlap similar to that shown in Fig. 2. The figure was prepared using Quantarctica 3.12 (www.npolar.no/quantarctica)^59^.

In contrast to male AFS, the fishery displayed different movement strategies that appeared to be related at least in part to gear type, though experience and choices made by the vessel Masters’ undoubtedly also play a role. CP trawlers alternated between the WAP and South Orkney Islands and most TR trawlers remained within the WAP but started the season much later (Fig. 4). Thus, while spatial overlap was apparent between male AFS and the fishery (and by extension, chinstrap penguins), the movement strategies of the fishery reduced temporal overlap. At the South Orkney Islands, the CP trawlers and male AFS overlapped temporally with almost the entire breeding season of chinstrap penguins, with fishing vessels leaving at the median date of chinstrap penguin fledging (Figs. 2, 4D). Temporal overlap in the WAP was more complex, with CP trawlers leaving and both male AFS and TR trawlers entering the area in mid-January (Fig. 4D). However, both fishing gear types and the majority of instrumented male AFS temporally and spatially overlapped the median chinstrap penguin fledging date (Figs. 2, 4D).

## Discussion

We provide the most detailed characterization of the spatiotemporal movements of male AFS and their overlap with the commercial krill fishery to date. While the seals occupied vast ranges compared to the fishery in 2016, areas used most intensively by male AFS overlapped with the fished areas. There was substantial temporal variability in the distribution of the two fishery predators, with the CP trawlers fishing off the western coast of the South Orkney Islands between January and March while the TR trawlers season started in mid-January and went through until June (Supplementary Fig. 1). In excess of 32,000 adult and subadult male AFS arrive in late January at Signy and Laurie Islands in the South Orkney Islands with recorded numbers decreasing throughout April^29,30^. The diet of male AFS at the South Orkney Islands is dominated by krill^31^; assuming an average age of 4 year^32^ and using the estimated amount of Antarctic krill consumed for this age class at South Georgia (3 t/year)^33^, the number of male AFS recorded at just these two sites in two months would consume approximately 24,000 t or 70% of the total recorded catch in Subarea 48.2 in 2016^34^. Areas intensively used by both male AFS and the fishery around the South Orkney Islands overlap in space and time with the late stages of chinstrap penguin breeding^35^ and numerous large penguin colonies occur along the western coastlines of the islands^36^. Importantly, the true number of male AFS arriving at the South Orkney Islands is likely to be substantially higher than that recorded at Signy and Laurie Islands alone, though abundances will likely vary considerably interannually^11^.

Our study shows that the spatial and temporal nature of potential resource competition between male AFS and the fishery and between both these groups and centrally foraging, krill dependent chinstrap penguins is likely to be complex. The male AFS study we conducted was concurrent with the second season of a similar tracking study on chinstrap penguins at the same location^18^, during one of the strongest El Ninos on record. Lowther et al.^18^ showed pronounced differences in chinstrap penguin foraging behaviour attributed to El Niño-driven coastal downwelling, forcing penguins to forage farther from shore in search of food presumably due to krill movement away into the open ocean. How similar events impact unconstrained predators such as male AFS is unknown, but even assuming that the patterns of space use we describe are consistent across years, the cumulative impacts of male AFS and fisheries on the foraging behaviour and reproductive success of chinstrap penguins at the South Orkney Islands is unlikely to be consistent because of the variability in numerical abundance of male AFS^11^ as well environmental stochasticity similar to that described by Lowther et al.^18^. Importantly, the degree and significance of the cumulative impacts of these various krill predators on each other is unknown, yet this understanding is important for appropriately interpreting the demographic trends of krill-dependent centrally foraging penguins. Presumably, the greatest impacts of competition on chinstrap penguins would be constrained to the western coastline of the South Orkney archipelago given the restricted foraging range of penguins during breeding^7^. Owing to the inaccessibility and lack of infrastructure in this area, there have been no meaningful population surveys of penguins in the last 40 years. However, future monitoring efforts should concentrate here given the potential for studying the direct impacts of fishing on centrally foraging penguins.

In the Bransfield and Gerlache Straits, the potential for resource competition and cumulative impacts of male AFS and the fishery on chinstrap penguins is even more complex. The core fishing area during penguin breeding in 2016 was located out of foraging range of brood and creche-stage chinstrap penguin breeding colonies on the South Shetlands, but within range of breeding colonies in the Gerlache Strait^6,37^. Only a small percentage of the total Subarea catch was taken during the breeding season, however the impact of the removals in terms of downstream effects on chinstrap penguin performance is unknown. Within the Gerlache Strait, only CP trawlers operated between December and mid-January, though their departure towards the east coincided with the arrival of the TR trawl fleet and male AFS.

Our study also highlighted the vertical component of spatial overlap between male AFS and the fishery. The diving behaviour of both male and female AFS has been characterized as deep dives giving way to shallower dives as daylight decreases, though these studies are typically conducted in the austral summer^38–40^. However, the WAP experiences near-total daylight during the summer, with no recordable civil twilight until the end of February and we show that male AFS dive to relatively shallow depths during summer daylight and only increase the depths and durations of their dives as daylight length shortened with the onset of winter. This switch in diving behaviour corresponded to a physical change in the water column structure that likely also signals a behavioural change in the vertical distribution of krill. As near-surface light-dependent phytoplankton abundance decreases, surface densities of krill also decrease and denser aggregations form at deeper depths (100–200 m)^41,42^. A similar change in the temporal distribution of fishing depth for both fishing gear types may reflect male AFS targeting similar densities of krill to those targeted by the fishery.

Regionally significant declines among chinstrap penguin populations have been identified throughout the WAP and are thought by some not to reflect direct impacts on breeding adults but rather to reflect declining post-fledging survival linked to decreases in krill abundance (or shifts in distribution)^43^. Interspecific competition with a suite of unconstrained, unmonitored and increasingly abundant krill predators such as male AFS, as well as changes in the vertical distribution of krill due to climactic forcing, may all be contributing to the penguin population declines. Adult pygoscelid penguins typically forage at shallow depths (30–40 m) during summer breeding^44^ but they can dive to depths in excess of 100 m^45^, which during the summer coincides with foraging depths used by male AFS in our study. After breeding, adult chinstrap penguins undertake large migrations away from breeding areas^46^ releasing them from trophic competition with male AFS and the fishery. However, it is currently impossible to estimate impacts on younger birds because the ontogeny of foraging behaviour both horizontally and vertically in fledgling pygoscelid penguins is poorly understood, though a pattern of progressively improving diving capabilities has been described for fledglings of other penguin species such as the Emperor penguins *Aptenodytes forsteri* and King penguin *A. patagonicus*^47^. Chinstrap fledglings depart their natal colonies in late February—early March^25,26^ and assuming they are initially shallower divers than adults, cumulative competition with male AFS and the fishery could impact their foraging efficiency and thus survival^48^. However, such interference competition would be conditional on spatial overlap between fledglings, male AFS and the fishery, the degree of which is currently unknown. Importantly though, in the context of interpreting penguin population demographic decline through reduced survival of a key life history stage, consideration should also be given to the likely nonlinear increase in potential competitive interactions, particularly given the rapid increase in male AFS abundance in recent decades^49^. Additional factors not directly related to this study should also be considered, such as the direct mortality of fledglings by male AFS with several studies highlighting the impact even a few penguin-eating seals can have^31,50^.

Through the treatment of fishing gear types as individual predator groups, our study also revealed very different spatiotemporal behaviours within the krill fishery. Continuous pumping trawlers occupied almost half of the fishing area used by TR trawlers between 2009 and 2018 (Table 2) yet caught between 56 and 66% of the catch over this period^51^. Our analysis of trawl depths over the season seems to indicate that CP trawlers target slightly deeper depths during the summer than their traditional counterparts until the onset of winter when both gear types fish at similar depths. Additionally, CP trawlers deploy and leave fishing gear deployed for prolonged periods and can maintain fishing effort at relatively low densities of krill^52^. Conversely, TR trawlers repeatedly set and haul trawl nets and try to fish in high densities to maintain production rates in the onboard factories^52^. Presumably the differences in operating depths, densities of krill and the level to which swarms can be economically depleted and duration that gear is deployed, particularly during the key summer breeding season for krill dependent predators, adds an additional (though currently unincorporated) layer of complexity to disentangling these interactions. Importantly, if CP trawlers are selected for in the future, there is currently no ability to predict how a shift in the dominant fishing gear type will change the impacts on the current suite of bioindicators such as those included in CEMP.

While the difference in core areas may reflect to some degree the different numbers of vessels operating each gear type and thus operational issues of safety leading to a spreading of vessel effort, the catch levels as a function of fishing area highlights the efficiency of the CP system^5^. In the WAP and Scotia Sea CCAMLR is faced with the difficult challenge of operationalising an adaptive EBFM system that responds to ecosystem information, and harmonizing this with the designation of a MPA with static management areas^9^. Extending the analogy of the fishery as a predator, one potential concern is an adaptive response, through a modification of fishing behaviour or patterns, to changes in the availability of prey resources as a result of static area-based management measures such as “no take” areas^53^. Studies examining the response of fisheries to management actions are limited, even though the impact of measures will profoundly affect how this “predator” adapts^53,54^. In the context of the krill fishery, a reduction in available fishing area may elicit a switch to more efficient gear such as continuous pumping, which in turn could increase the risk of localized depletion^13^. However, managing the fishery adaptively at the scale at which it operates is feasible, given that a substantial portion of the vessels within the krill fishing fleet are fitted with scientific-quality acoustic echosounders that have proven capable of providing near-real-time estimates of krill abundance and distribution in an area being fished^55–57^. Incorporating such information into fisheries assessments might provide an opportunity to adaptively scale catch to local krill availability, preventing localized depletion.

We demonstrate that male AFS, whose abundance has increased rapidly throughout the lifetime of the Antarctic krill fishery, and on which monitoring effort is lacking, overlaps in all four dimensions (horizontal, vertical and temporal) with the krill fishery in a complex mosaic. We show that the fishery itself is comprised of two distinct predator types, with the more modern and efficient CP trawlers possibly being selected for in the future should static management efforts constrict the area over which the fishery can operate, fuelling concerns over localised depletion. Together, male AFS and the krill fishery likely contribute to cumulative impacts on centrally foraging predators monitored in CEMP. A holistic view will provide not only a more detailed understanding of the impact of the fishery, but also its interaction with species on the breeding and recruitment success of CEMP monitored species.

However, sophisticated, spatially resolved modelling approaches have already been proposed to evaluate the demographic trends of penguins throughout the WAP and Scotia Sea. These models are better equipped to capture the complexity of cumulative, spatially heterogeneous competitive interactions between species and the nonlinear effects of climate change^58^, and may lead to a more transparent and implementable EBFM system for the Antarctic krill fishery.

## Supplementary information

Supplementary Figure 1.

Supplementary Figure 2.

Supplementary Figure 3.

Supplementary Figure 4.

Supplementary Table 1.

## References

[1] Zhou S (2010). Ecosystem-based fisheries management requires a change to the selective fishing philosophy. Proc. Natl. Acad. Sci. U.S.A..

[2] Fulton EA, Smith ADM, Smith DC, Johnson P (2014). An integrated approach is needed for ecosystem based fisheries management: insights from ecosystem-level management strategy evaluation. PLoS ONE.

[3] Murphy EJ (2016). Understanding the structure and functioning of polar pelagic ecosystems to predict the impacts of change. Proc. R. Soc. B Biol. Sci..

[4] Schofield O (2018). Changes in the upper ocean mixed layer and phytoplankton productivity along the West Antarctic Peninsula. Philos. Trans. R. Soc. A Math. Phys. Eng. Sci..

[5] Nicol S, Foster J, Kawaguchi S (2012). The fishery for Antarctic krill-recent developments. Fish Fish..

[6] Trathan PN (2018). Managing fishery development in sensitive ecosystems: identifying penguin habitat use to direct management in Antarctica. Ecosphere.

[7] Warwick-Evans V (2018). Using habitat models for chinstrap penguins *Pygoscelis antarctica* to advise krill fisheries management during the penguin breeding season. Divers. Distrib..

[8] Santa Cruz F, Ernst B, Arata JA, Parada C (2018). Spatial and temporal dynamics of the Antarctic krill fishery in fishing hotspots in the Bransfield Strait and South Shetland Islands. Fish. Res..

[9] Sylvester ZT, Brooks CM (2020). Protecting Antarctica through Co-production of actionable science: lessons from the CCAMLR marine protected area process. Mar. Policy.

[10] Agnew DJ (1997). Review: the CCAMLR ecosystem monitoring programme. Antarct. Sci..

[11] Boyd IL, McCafferty DJ, Reid K, Taylor R, Walker TR (1998). Dispersal of male and female antarctic fur seals (*Arctocephalus gazella*). Can. J. Fish. Aquat. Sci..

[12] Vergani DF, Coria NR (1989). Increase in numbers of male fur seals *Arctocephalus gazella* during the summer autumn period at Mossman Peninsula (Laurie Island). Polar Biol..

[13] Plagányi ÉE, Butterworth DS (2012). The Scotia Sea krill fishery and its possible impacts on dependent predators: modeling localized depletion of prey. Ecol. Appl..

[14] Watters GM, Hill SL, Hinke JT, Matthews J, Reid K (2013). Decision-making for ecosystem-based management: evaluating options for a krill fishery with an ecosystem dynamics model. Ecol. Appl..

[15] Trivelpiece WZ (2011). Variability in krill biomass links harvesting and climate warming to penguin population changes in Antarctica. Proc. Natl. Acad. Sci. U.S.A..

[16] Watters GM, Hinke JT, Reiss CS (2020). Long-term observations from Antarctica demonstrate that mismatched scales of fisheries management and predator–prey interaction lead to erroneous conclusions about precaution. Sci. Rep..

[17] Hill SL, Reid K, Thorpe SE, Hinke J, Watters GM (2007). A compilation of parameters for ecosystem dynamics models of the Scotia Sea—Antarctic Peninsula region. CCAMLR Sci..

[18] Lowther AD, Trathan P, Tarroux A, Lydersen C, Kovacs KM (2018). The relationship between coastal weather and foraging behaviour of chinstrap penguins *Pygoscelis antarctica*. ICES J. Mar. Sci..

[19] Boehme L (2009). Technical note: animal-borne CTD-satellite relay data loggers for real-time oceanographic data collection. Ocean Sci..

[20] Lowther AD, Lydersen C, Fedak MA, Lovell P, Kovacs KM (2015). The Argos-CLS Kalman filter: error structures and state-space modelling relative to Fastloc GPS data. PLoS ONE.

[21] Johnson DS, London JM, Lea MA, Durban JW (2008). Continuous-time correlated random walk model for animal telemetry data. Ecology.

[22] Benhamou S (2011). Dynamic approach to space and habitat use based on biased random bridges. PLoS ONE.

[23] Schlitzer, R. Ocean Data View. https://odv.awi.de (2018).

[24] Hill SL, Trathan PN, Agnew DJ (2009). The risk to fishery performance associated with spatially resolved management of Antarctic krill (*Euphausia superba*) harvesting. ICES J. Mar. Sci..

[25] Trivelpiece WZ, Trivelpiece SG, Volkman NJ (1987). Ecological segregation of Adelie, gentoo, and chinstrap penguins at King George Island Antarctica. Ecology.

[26] Black CE (2016). A comprehensive review of the phenology of *Pygoscelis penguins*. Polar Biol..

[27] Payne MR (1979). Growth in the Antarctic fur seal *Arctocephalus gazella*. J. Zool..

[28] Reiss CS (2017). Overwinter habitat selection by Antarctic krill under varying sea-ice conditions: implications for top predators and fishery management. Mar. Ecol. Prog. Ser..

[29] Carlini AR, Daneri GA, Casaux R, Márquez MEI (2006). Haul-out pattern of itinerant male Antarctic fur seals (*Arctocephalus gazella*) at Laurie Island South Orkney Islands. Polar Res..

[30] Waluda CM, Gregory S, Dunn MJ (2010). Long-term variability in the abundance of Antarctic fur seals *Arctocephalus gazella* at Signy Island South Orkneys. Polar Biol..

[31] Casaux R, Juares M, Carlini A, Corbalán A (2016). The diet of the Antarctic fur seal *Arctocephalus gazella* at the South Orkney Islands in ten consecutive years. Polar Biol..

[32] Reid K, Arnould JPYY (1996). The diet of Antarctic fur seals *Arctocephalus gazella* during the breeding season at South Georgia. Polar Biol..

[33] Boyd IL (2002). Estimating food consumption of marine predators: Antarctic fur seals and macaroni penguins. J. Appl. Ecol..

[34] CCAMLR. *Krill Fishery Report*. *Commission for the Conservation of Antarctic Marine Living Resources*. https://www.ccamlr.org/en/system/files/00%20KRI48%202016%20v1_1.pdf (2016).

[35] Dias MP (2018). Identification of marine important bird and biodiversity areas for penguins around the South Shetland Islands and South Orkney Islands. Ecol. Evol..

[36] Lynch HJ, Naveen R, Casanovas P (2013). Antarctic site inventory breeding bird survey data, 1994–2013. Ecology.

[37] Humphries GRW (2017). Mapping application for penguin populations and projected dynamics (MAPPPD): data and tools for dynamic management and decision support. Polar Rec. (Gr. Brit).

[38] Lea MA (2008). Colony-based foraging segregation by Antarctic fur seals at the Kerguelen Archipelago. Mar. Ecol. Prog. Ser..

[39] Staniland IJ, Reid K, Boyd IL (2004). Comparing individual and spatial influences on foraging behaviour in Antarctic fur seals *Arctocephalus gazella*. Mar. Ecol. Prog. Ser..

[40] Bonadonna F, Lea MA, Guinet C (2000). Foraging routes of Antarctic fur seals (*Arctocephalus gazella*) investigated by the concurrent use of satellite tracking and time-depth recorders. Polar Biol..

[41] Richerson K, Santora JA, Mangel M (2017). Climate variability and multi-scale assessment of the krill preyscape near the north Antarctic Peninsula. Polar Biol..

[42] Cleary AC, Durbin EG, Casas MC, Zhou M (2016). Winter distribution and size structure of Antarctic krill *Euphausia superba* populations in-shore along the West Antarctic Peninsula. Mar. Ecol. Prog. Ser..

[43] Lynch HJ, Naveen R, Trathan PN, Fagan WF (2012). Spatially integrated assessment reveals widespread changes in penguin populations on the Antarctic Peninsula. Ecology.

[44] Kokubun N, Takahashi A, Mori Y, Watanabe S, Shin HC (2010). Comparison of diving behavior and foraging habitat use between chinstrap and gentoo penguins breeding in the South Shetland Islands Antarctica. Mar. Biol..

[45] Miller AK, Trivelpiece WZ (2008). Chinstrap penguins alter foraging and diving behavior in response to the size of their principle prey Antarctic krill. Mar. Biol..

[46] Hinke JT, Santos MM, Korczak-Abshire M, Milinevsky G, Watters GM (2019). Individual variation in migratory movements of chinstrap penguins leads to widespread occupancy of ice-free winter habitats over the continental shelf and deep ocean basins of the Southern Ocean. PLoS ONE.

[47] Orgeret F, Weimerskirch H, Bost CA (2016). Early diving behaviour in juvenile penguins: improvement or selection processes. Biol. Lett..

[48] Marchetti K, Price T (1989). Differences in the foraging of juvenile and adult birds: the importance of developmental constraints. Biol. Rev. Camb. Philos. Soc..

[49] Boyd IL (1993). Pup production and distribution of breeding antarctic fur seals (*Arctocephalus gazella*) at South Georgia. Antarct. Sci..

[50] Casaux R (2003). Geographical variation in the diet of the Antarctic fur seal *Arctocephalus gazella*. Polar Biol..

[51] CCAMLR. *Statistical Bulletin.* Vol. 31. www.ccamlr.org (2019).

[52] Kawaguchi S, Nicol S (2020). Krill fishery. Fish. Aquac..

[53] Vaughan D (2017). Fishing effort displacement and the consequences of implementing marine protected area management: an English perspective. Mar. Policy.

[54] Suuronen P, Jounela P, Tschernij V (2010). Fishermen responses on marine protected areas in the Baltic cod fishery. Mar. Policy.

[55] Godø OR, Reiss C, Siegel V, Watkins JL (2014). Commercial fishing vessel as research vessels in the Antarctic: requirements and solutions exemplified with a new vessel. CCAMLR Sci..

[56] Niklitschek EJ, Skaret G (2016). Distribution, density and relative abundance of Antarctic krill estimated by maximum likelihood geostatistics on acoustic data collected during commercial fishing operations. Fish. Res..

[57] Watkins JL (2016). The use of fishing vessels to provide acoustic data on the distribution and abundance of Antarctic krill and other pelagic species. Fish. Res..

[58] Melbourne-Thomas J, Constable A, Wotherspoon S, Raymond B (2013). Testing paradigms of ecosystem change under climate warming in Antarctica. PLoS ONE.

[59] Matsuoka, K., Skoglund, A. & Roth, G. Quantarctica [Data set]. Norwegian Polar Institute. 10.21334/npolar.2018.8516e961 (2018).

