# Supplementary material for: Male Antarctic fur seals: neglected food competitors of bioindicator species in the context of an increasing Antarctic krill fishery
Source: Sci Rep. 2020 Oct 28;10:18436. doi: 10.1038/s41598-020-75148-9 (PMC7595138; doi:10.1038/s41598-020-75148-9)
Supplement: Supplementary file 1 — Supplementary Figure 1. [file 41598_2020_75148_MOESM1_ESM.pdf]

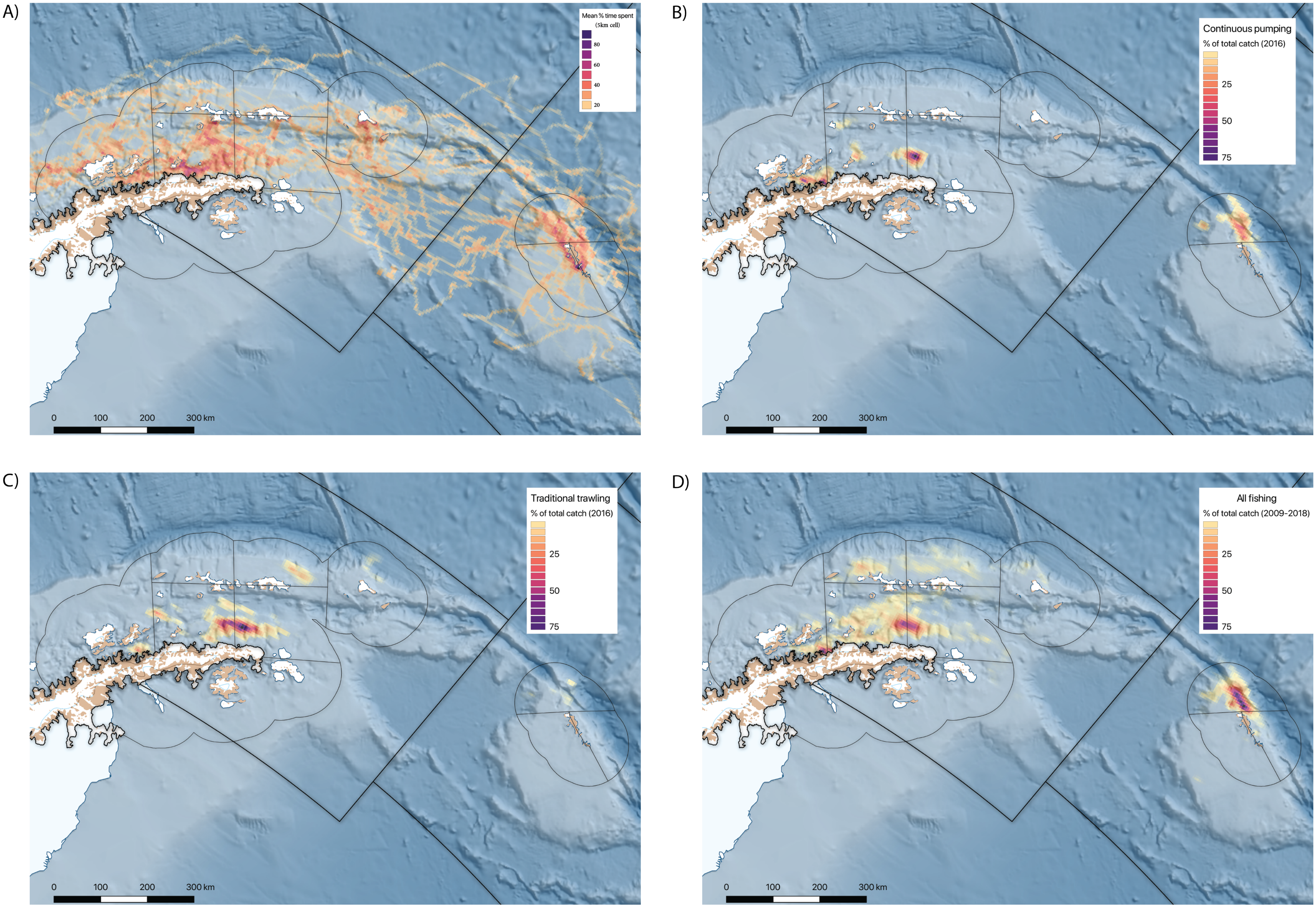

Supplementary Fig. 1: A) Mean time spent in 5km resolution grid cells by male Antarctic fur seals (N=18) instrumented in January 2016 at the South Orkney Islands, relative to the proportion of total catch for B) Continuous pumping trawlers and C) Traditional trawlers in 2016 and D) the entire fishery between 2009 and 2018. The figure was created using Quantarctica 3.12 (www.npolar.no/quantarctica) [59]
