# Supplementary material for: Male Antarctic fur seals: neglected food competitors of bioindicator species in the context of an increasing Antarctic krill fishery
Source: Sci Rep. 2020 Oct 28;10:18436. doi: 10.1038/s41598-020-75148-9 (PMC7595138; doi:10.1038/s41598-020-75148-9)
Supplement: Supplementary file 2 — Supplementary Figure 2. [file 41598_2020_75148_MOESM2_ESM.pdf]

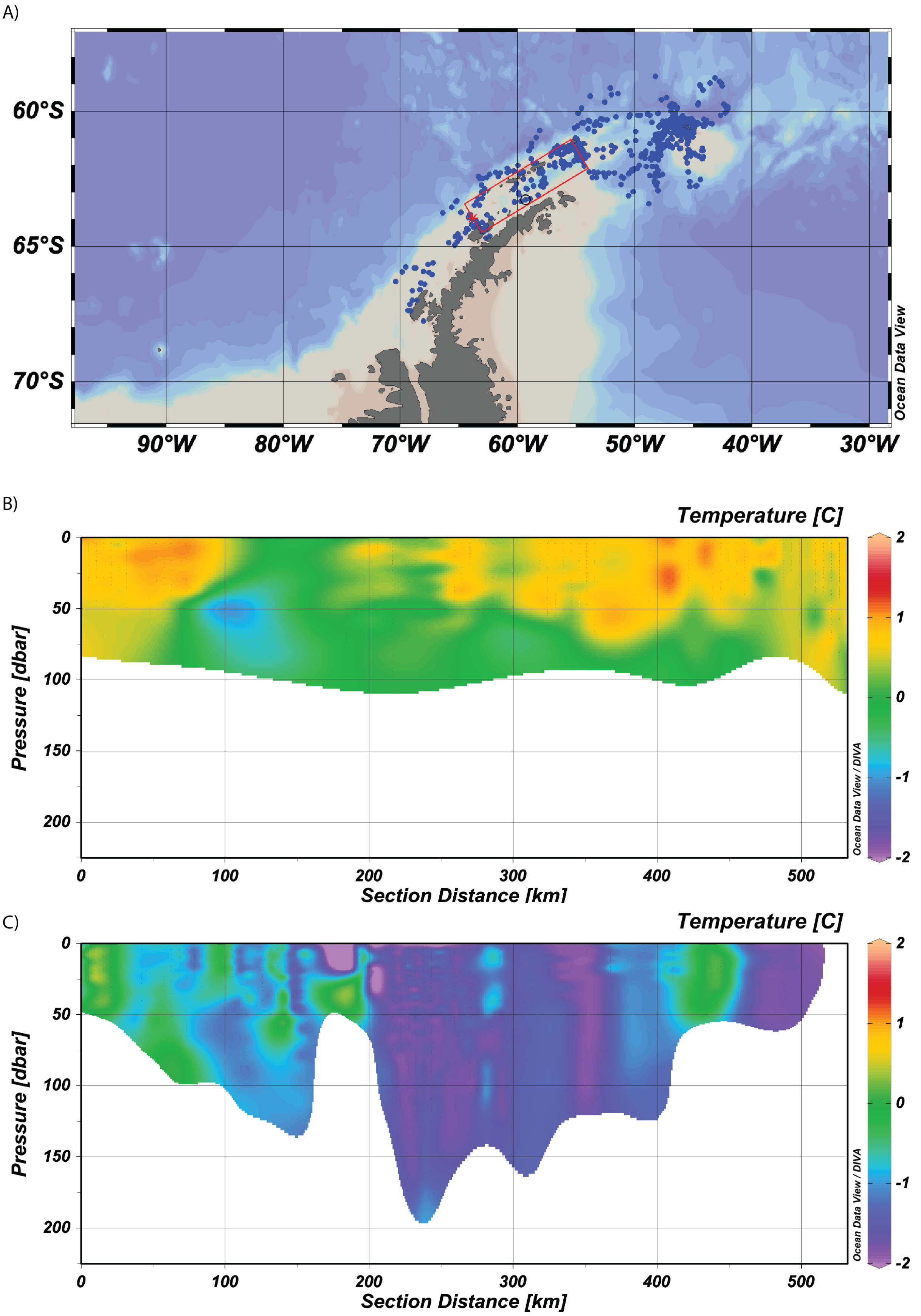

Supplementary Fig. 4: Temperature profiles from SRDL-instrumented adult male Antarctic fur seals. A) A 500km-wide section running east to west along the length of the Bransfield and Gerlache Straits was used to derive a three-dimensional empirical representation of the water column between B) January and March (summer) and C) April and May (autumn / early winter). During summer the upper 100m of the Bransfield Strait was relatively warm, reaching between 0 and 2 degrees celcius. Through late autumn and the onset of winter these temperatures cooled, coinciding with more diffuse diving strategies of4 adult male Antarctic fur seals (Fig 3).The figure was created using Ocean Data View (ODV 4.7.8) www.odv.awi.de [23]
