# Supplementary material for: Male Antarctic fur seals: neglected food competitors of bioindicator species in the context of an increasing Antarctic krill fishery
Source: Sci Rep. 2020 Oct 28;10:18436. doi: 10.1038/s41598-020-75148-9 (PMC7595138; doi:10.1038/s41598-020-75148-9)
Supplement: Supplementary file 3 — Supplementary Figure 3. [file 41598_2020_75148_MOESM3_ESM.pdf]

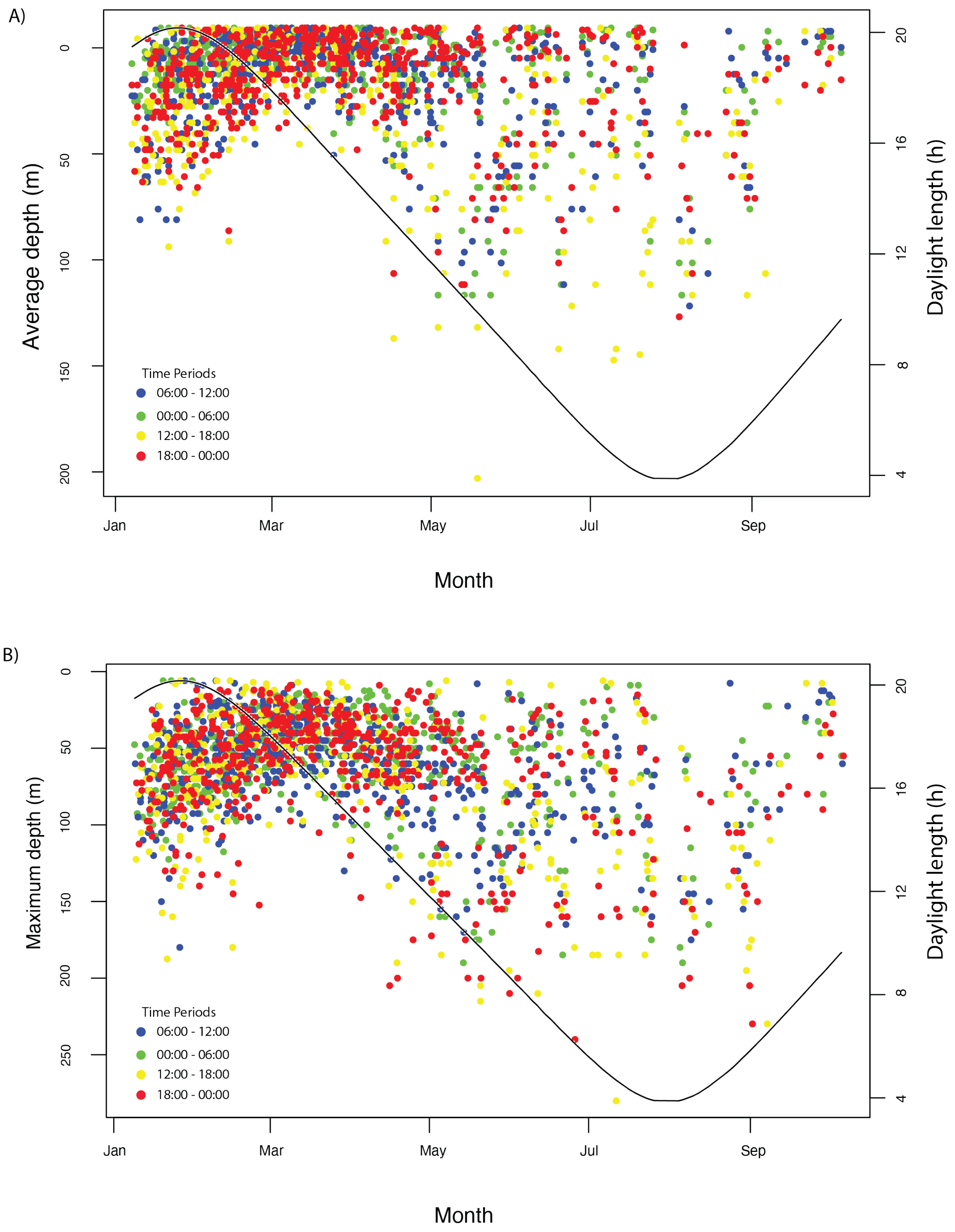

Supplementary Fig. 2: Dive summary data for 20 adult male Antarctic fur seals instrumented between January and October 2016 at Powell Island in the South Orkney Islands archipelago. Points represent the mean estimate of A) average dive depths and B) maximum dive depths recorded in consecutive 6 hour time periods (00:00-06:00, 06:00-12:00, 12:00-18:00, 18:00 - 00:00). Note the different depth scales. Black curvilinear lines represent daylight length (h) calculated at King George Island. Both average and maximum dive depths were much shallower between January and March, becoming deeper as daylight length decreased into winter..
