# Supplementary material for: Male Antarctic fur seals: neglected food competitors of bioindicator species in the context of an increasing Antarctic krill fishery
Source: Sci Rep. 2020 Oct 28;10:18436. doi: 10.1038/s41598-020-75148-9 (PMC7595138; doi:10.1038/s41598-020-75148-9)
Supplement: Supplementary file 4 — Supplementary Figure 4. [file 41598_2020_75148_MOESM4_ESM.pdf]

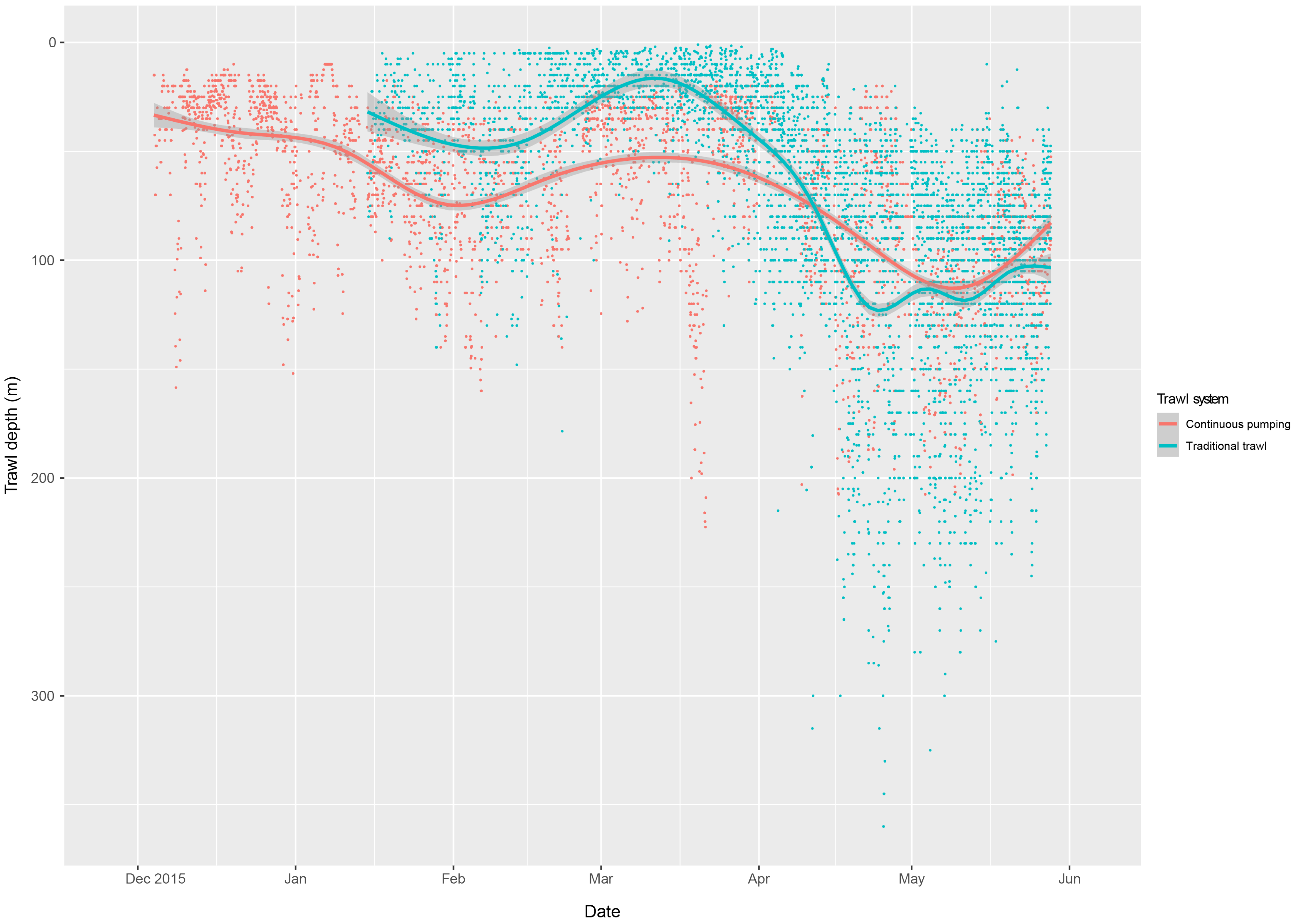

Supplementary Fig. 3: Trawl Depths throughout the 2016 fishing season for Continuous Pump and Traditional Trawl vessels. Note the decreasing depth of fishing through the progression to winter, consistent to that seen in male Antarctic fur seal diving behaviour (Supplementary Fig 2).
