# Supplementary material for: Male Antarctic fur seals: neglected food competitors of bioindicator species in the context of an increasing Antarctic krill fishery
Source: Sci Rep. 2020 Oct 28;10:18436. doi: 10.1038/s41598-020-75148-9 (PMC7595138; doi:10.1038/s41598-020-75148-9)
Supplement: Supplementary file 5 — Supplementary Table 1. [file 41598_2020_75148_MOESM5_ESM.pdf]

Supplementary Table 1: Summarised chemical immobilisation data for 20 adult male Antarctic fur seals instrumented with either low profile Satellite Relay Data Loggers (SRDL) with integrated dive loggers (N=18) or Conductivity-Temperature-Depth SRDL (N=2) between 5th and 26th January 2016 at Powell Island, South Orkney Islands. Dosage rates are for Zoletil adminstration with Isofluorane provided at 5% induction and 1-3% maintenance. Times are given for induction using a combination of Zoletil and Isofluorane, with missing values indicating either that Zoletil was not administered or Isofluorane was not required.

ID	Date	Body Length (cm)	Dosage (ml/kg)	Induction time (min)		Duration (min)	
				Zoletil	Isofluorane	Procedure	Recovery
ag01-01-15	05/01/2016	171.0	1.50	9	9	15	48
ag01-02-15	06/01/2016	182.0	1.40	10	-	22	19
ag01-03-15	06/01/2016	171.0	1.50	6	-	16	53
ag01-04-15	07/01/2016	160.0	1.40	9	-	16	4
ag01-06-15	08/01/2016	160.5	1.40	10	3	23	50
ag01-07-15	08/01/2016	166.5	1.50	10	5	21	9
ag01-08-15	09/01/2016	172.5	1.50	27	2	17	5
ag01-09-15	09/01/2016	166.5	1.40	9	2	15	24
ag01-10-15	10/01/2016	156.0	1.50	11	5	15	45
ag01-11-15	10/01/2016	171.0	1.50	19	-	12	47
ag01-12-15	11/01/2016	174.0	1.50	12	-	20	2
ag01-13-15	11/01/2016	184.0	1.40	8	5	21	14
ag01-14-15	21/01/2016	140.0	0.45	-	22	19	15
ag01-16-15	21/01/2016	143.5	0.50	-	9	23	19
ag01-17-15	21/01/2016	134.0	0.40	-	9	18	1
ag01-18-15	21/01/2016	127.0	0.40	-	9	23	13
ag01-19-15	23/01/2016	140.0	0.30	-	11	25	1
ag01-20-15	23/01/2016	150.0	0.40	-	7	21	18
ag01-244g-14	26/01/2016	175.0	1.80	9	6	17	19
ag01-273-14	26/01/2016	175.0	1.80	13	-	18	27
